# Evaluation of the probiotic potential of yeast isolated from kombucha in New Zealand

**DOI:** 10.1016/j.crfs.2024.100711

**Published:** 2024-03-08

**Authors:** Boying Wang, Kay Rutherfurd-Markwick, Ninghui Liu, Xue-Xian Zhang, Anthony N. Mutukumira

**Affiliations:** a School of Food and Advanced Technology, Massey University, Auckland, 0745, New Zealand; b School of Health Sciences, Massey University, Auckland, 0745, New Zealand; c School of Natural Sciences, Massey University, Auckland, 0745, New Zealand

**Keywords:** Kombucha, Fermentation, Probiotics, Yeast

## Abstract

The current study investigated the *in vitro* probiotic potential of yeast isolated from kombucha, a tea beverage fermented with a symbiotic culture of acetic acid bacteria and yeast. A total of 62 yeast strains were previously isolated from four different commercial kombucha samples sold in New Zealand. Fifteen representative isolates belonging to eight different species were evaluated for their growth under different conditions (temperature, low pH, concentrations of bile salts, and NaCl). Cell surface characteristics, functional and enzymatic activities of the selected strains were also studied in triplicate experiments. Results showed that six strains (*Dekkera bruxellensis* LBY1, *Sachizosaccharomyces pombe* LBY5, *Hanseniaspora valbyensis* DOY1, *Brettanomyces anomalus* DOY8, *Pichia kudraivzevii* GBY1, and *Saccharomyces cerevisiae* GBY2) were able to grow under low-acid conditions (at pH 2 and pH 3) and in the presence of bile salts. This suggests their potential to survive passage through the human gut. All 15 strains exhibited negative enzymatic activity reactions (haemolytic, gelatinase, phospholipase, and protease activities), and thus, they can be considered safe to consume. Notably, two of the fifteen strains (*Pichia kudraivzevii* GBY1 and *Saccharomyces cerevisiae* GBY2) exhibited desirable cell surface hydrophobicity (64.60–83.87%), auto-aggregation (>98%), co-aggregation, resistance to eight tested antibiotics (ampicillin, chloramphenicol, colistin sulphate, kanamycin, nalidixic acid, nitrofurantoin, streptomycin, and tetracycline), and high levels of antioxidant activities (>90%). Together, our data reveal the probiotic activities of two yeast strains GBY1 and GBY2 and their potential application in functional food production.

## Highlights

• Fifteen representative yeast strains from commercial Kombucha samples sold in New Zealand were evaluated for their *in vitro* probiotic potential.
• *Pichia kudraivzevii* GBY1 and *Saccharomyces cerevisiae* GBY2 exhibited excellent antioxidant and probiotic activities.
• Data consistently show that GBY1 and GBY2 are potential probiotics suitable for application in functional foods.

## Graphical abstract

**Figure:**
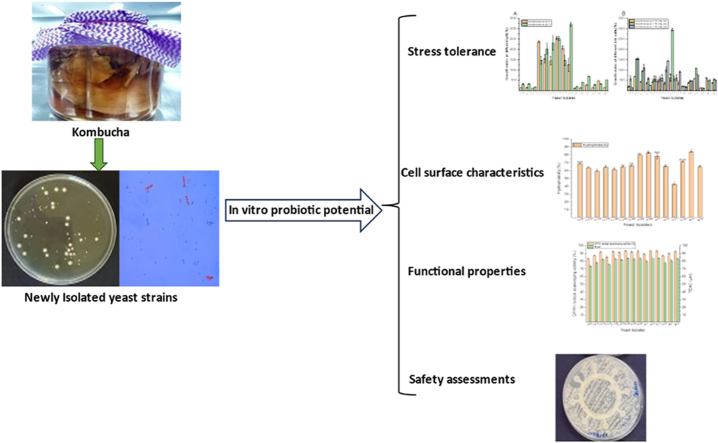

## 1 Introduction

Kombucha is a popular, slightly sweet, sparkling drink fermented for 7–10 days at ambient temperature in a base of sugared tea infusion, typically made with black or green tea (de Miranda et al., 2022; Jayabalan et al., 2014; Nyhan et al., 2022). The fermentation is conducted by a complex symbiotic culture of acetic acid bacteria (AAB) and yeast, commonly known as SCOBY. Some lactic acid bacteria (LAB) have been reported in fermented kombucha, however, available information suggests that they may not be essential for the fermentation of the beverage (Laureys et al., 2020). Regular consumption of kombucha may confer beneficial health-promoting effects, including antioxidant, anti-inflammatory, antibacterial, probiotic and anticarcinogenic activities (Coelho et al., 2020; Jayabalan et al., 2014; Jayabalan and Waisundara, 2019; Selvaraj and Gurumurthy, 2022). Due to the appealing sensory characteristics and beneficial effects, kombucha has gained popularity around the world (Batista et al., 2022; Kapp and Sumner, 2019). Kombucha was reported to have a global market of about USD 1.5 billion around 2018, and the market value of fermented beverage is predicted to increase to 5 billion by 2025 (Kim and Adhikari, 2020).

The microbial composition of kombucha starter cultures is diverse and complex, and it is largely determined by the original sources of the SCOBY and the cultivation conditions (Akhtar et al., 2021; Jayabalan et al., 2014; Mayser et al., 1995). Acetic acid bacteria in kombucha are dominated by *Komagataeibacteri xylinus*, *K. rhaeticus, Acetobacter aceti, A. tropicalis, A. pasteurianus, Gluconobacter oxydans and Gluconacetobacter sacchari* (Akhtar et al., 2021; Barbosa et al., 2021; Jayabalan et al., 2014; Pradhan et al., 2022; Savary et al., 2021; Semjonovs et al., 2017; Wang et al., 2022b, 2023). The yeast community is generally more diverse than AAB, and it includes *Zygosaccharomyces bailii, Z. rouxii, Brettanomyces lambrcus, B. custerisii, B. intermedius, B. claussenii, B. bruxellensis Candida albican, C. kefir, C. obtuse, Schizosaccharomyces pombe, Pichia fermentans, P. membranefaciens, Torulaspora delbrueckii, T. famata* and *Saccharomyces cerevisiae* (Arikan et al., 2020; Chakravorty et al., 2016; Gaggia et al., 2018; Jayabalan et al., 2014; Marsh et al., 2014; Mayser et al., 1995; Pradhan et al., 2022; Ramadani and Abulreesh, 2010; Teoh et al., 2004; Wang et al., 2022a). The presence of these microorganisms may be associated with the reported probiotic characteristics of kombucha.

Probiotics are defined as live microorganisms with appropriate concentrations of well-defined strains which exhibit health-promoting benefits to the host (Hill et al., 2014). To confer health benefits, probiotic microorganisms must be able to survive in the dynamic gastrointestinal environment, which includes low pH and pepsin in the stomach and bile salts and pancreatic enzymes in the intestine. Additionally, they must be capable of propagating at human body temperatures (Gil-Rodríguez et al., 2015; Hossain et al., 2020; Hsu and Chou, 2021; Suvarna et al., 2018). Therefore, several selection criteria are essential for microorganisms to be considered as probiotics, including being safe for consumption and producing non-toxic activities to the human body (Lahtinen et al., 2009). A safety assessment of any potential probiotic strain must be conducted before the culture can be considered for use in food or pharmaceutical products (FAO/WHO, 2006; Sanders et al., 2010). The ability of probiotics to adhere to epithelial cells is a desirable characteristic of beneficial microorganisms allowing them to manifest their beneficial effects by persisting longer in the host GI tract (Collado et al., 2008; García-Cayuela et al., 2014). Antioxidant and antimicrobial activities have also been used to identify potential probiotic strains (Binetti et al., 2013; Chen et al., 2010; Liu et al., 2021).

Most probiotics are lactic acid bacteria (LAB) with the majority belonging to the genera *Lactobacillus, Enterococcus and Bifidobacterium* (Alvarez et al., 2023; Kim et al., 2022; Liu et al., 2021). However, to date only a few yeast strains have been identified as probiotics (Suvarna et al., 2018). *Sacchromyces cerevisiae* and *S. boulardii* are the only commercial probiotic yeast species currently available for human consumption (Di Cagno et al., 2020; Suvarna et al., 2018). Yeast is commonly found in food and beverages and are utilised as starter cultures for fermentation of several food and beverages such as wine, kombucha, lambic beer, kefir, sake, bread-making, and table olives (Alkalbani et al., 2022a,b). Their wide applications in the food industry indicate that most food-related fungi (yeast) are generally regarded as safe (Hsu and Chou, 2021). Recently, consideration of yeast as potential probiotic microorganisms has increased due to their advantages over bacteria (Alvarez et al., 2023), for example, the larger size of yeast cells (approximately 10 times larger) compared to bacteria allow them to exhibit stearic hindrance to bacteria (Czerucka et al., 2007). Yeast such as the genera *Pichia, Schizosaccharomyces, Kluyveromyces, Yarrow,* and *Torulaspora* have shown several beneficial characteristics for health, including excellent resistance to antibiotics which helps to restore gut microbiota after antibiotic administration. Yeast is also characterised by having a high mineral and vitamin B content, the presence of several immune response components in their cell wall such as mannose and glucan, and ability to grow under conditions similar to the harsh GI tract environment (Czerucka et al., 2007; Gil-Rodríguez et al., 2015; Li et al., 2020). Despite a large number of probiotics being used in commercial products, the rapid growth of the probiotic market indicates that demand exists for more strains possessing specific functional properties such as antioxidant and anti-inflammatory activities (Pereira et al., 2021). Therefore, it is necessary to determine the characteristics of other potential probiotic yeast species or strains from food or beverage sources (Alvarez et al., 2023; Hsu and Chou, 2021).

Kombucha is generally regarded as a probiotic drink due its inherent live microorganisms (Akhtar et al., 2021; Vargas et al., 2021). Although many commercial kombucha products are labelled as containing “live cultures”, their probiotic properties have not been documented (Kim and Adhikari, 2020; Vargas et al., 2021). At present, there is scanty information on the relative abundance of the viable microorganisms in kombucha and their perceived health promoting properties (Laureys et al., 2020; Vargas et al., 2021). Here we report the evaluation of the *in vitro* probiotic potential of 15 representative yeast strains previously isolated from commercial kombucha samples produced in New Zealand.

## 2 Material and methods

### 2.1 Yeast strains and cultivation conditions

Sixty-two yeast strains from eight species were isolated from three branded kombucha beverages purchased from local supermarkets in Auckland, New Zealand. The cultures were isolated from the kombucha broth and bacterial cellulose (SCOBY). The identities of the yeast strains were reported earlier (Wang et al., 2022a, 2023). Of the 62 strains isolated, 15 representative yeast isolates were chosen and differentiated based on their phenotypic and biochemical characteristics and survival during fermentation (Wang et al., 2022a, 2023). All 15 representative yeast isolates **(**Table 1**)** were preserved in 20% glycerol (w/w) and stored at −80 °C until required. Before use, the frozen cultures were activated on potato dextrose agar (PDA) plates for 5 days or yeast peptone broth (YPD) overnight.

**Table 1:** Identities of yeast strains and their origins.

Specie	Strain	Source
*Debaryomyces prosopidis*	^1^D3Y9	Kombucha tea broth
*Debaryomyces prosopidis*	^1^D5Y12	Kombucha tea broth
*Debaryomyces prosopidis*	^1^D7Y18	Kombucha tea broth
*Zygosaccharomyces lentus*	^1^D7T19	Kombucha tea broth
*Zygosaccharomyces lentus*	^1^D11Y30	Kombucha tea broth
*Zygosaccharomyces lentus*	^1^D14Y35	Kombucha tea broth
*Zygosaccharomyces lentus*	^1^TFY36	Cellulosic pellicle
*Zygosaccharomyces lentus*	^1^TFY38	Cellulosic pellicle
*Zygosaccharomyces lentus*	^1^TFY39	Cellulosic pellicle
*Dekkera bruxellensis*	^2^LBY1	Kombucha brand 1
*Sachizosaccharomyces pombe*	^2^LBY5	Kombucha brand 1
*Hanseniaspora valbyensis*	^3^DOY1	Kombucha brand 2
*Brettanomyces anomalus*	^3^DOY8	Kombucha brand 2
*Pichia kudraivzevii*	^4^GBY1	Kombucha brand 3
*Saccharomyces cerevisiae*	^4^GBY2	Kombucha brand 3

Note: ^1^strains were selected from beginning, middle and end of fermentations from SCOBY. ^2,3,4^ yeast isolates were selected based on representative strains of each species with the same phenotypic characteristics.

### *2.2* *In vitro* tolerance of yeast strains to simulated gastrointestinal tract (GIT) conditions

#### 2.2.1 Tolerance to low pH

The tolerance of each yeast strain to low pH was determined using the method of Hsu and Chou (2021) with minor modifications. Briefly, overnight suspensions (1%, v/v) of each yeast strain were inoculated into YPD broth tubes adjusted to pH 2.0 and pH 3.0 with 0.1M HCl (Sigma Aldrich, St. Louis, MO, USA). The inoculated cultures were incubated at 25 °C for 24 h. Growth of yeast cultures was confirmed by measuring the absorbance of grown cultures at 600 nm using a spectrophotometer (Novaspec III, Amersham Biosciences, Buckinghamshire, United Kingdom). The non-inoculated YPD broths (pH 2 and pH 3) were calibrated as the control blanks respectively and the growth index was calculated as shown in equation [1].

[1] $$\text{Growth}\;\text{index}\left(\%\right)=\left(\frac{A_{600\;}\left(24h\right)}{A_{600}\left(0h\right)}\right)*100\%$$

A_600_ (0h): Absorbance_600nm_ at 0h
A_600_(24h): Absorbance_600nm_ at 24h

#### 2.2.2 Tolerance to bile salts

Tolerance of cultures to bile salts was carried out using the method of Bogdan et al. (2018). Overnight suspensions (1%, v/v) of each yeast strain were inoculated into YPD broth supplemented with 0.5%, 1.0% and 1.5% (w/v) bile salts (Sigma Aldrich) and incubated at 25 °C for 24 h (Merchán et al., 2020). Absorbance of the incubated cultures were measured at 0 h and 24 h in a spectrophotometer at 600 nm to determine their growth. The non-inoculated YPD broth with different bile salt concentrations was calibrated as the control blank and the growth index was calculated using equation [1].

#### 2.2.3 Growth at different temperatures

The growth of yeast at different temperatures was examined using the method of Alvarez et al. (2023) with some modifications. Loopfuls of overnight yeast isolate were streaked on PDA plates and incubated at the selected temperatures: 25 °C and 30 °C (kombucha fermentation temperature), 37 °C (human internal body temperature and anaerobic conditions), 39 °C and 42 °C (fever temperature) for 5 days. The development of colonies along the streaked lines was indicative of growth and therefore deemed a positive result.

#### 2.2.4 Tolerance to NaCl_aqueous_

The growth of yeast isolates under different salt (NaCl) concentrations was examined using the method of Zeng et al. (2019) with modifications. The PDA plates were supplemented with NaCl (2%, 4%, 6.5%, w/v). Loopfuls of each overnight cultured yeast strain were streaked on PDA plates with different salt concentrations and incubated at 25 °C for 5 days. The formation of colonies along the streaked lines indicated growth and tolerance to NaCl.

### 2.3 Cell surface characteristics

#### 2.3.1 Auto-aggregation and co-aggregation assay

Determination of auto-aggregation was performed according to Gil-Rodríguez et al. (2015) with some modifications. Each yeast strain was separately cultured in YPD broth overnight. The cell pellets were harvested by centrifugation at 8000*g* for 5 min at 4 °C, washed twice with sterile 0.9% NaCl solution and resuspended in the same sterile saline solution-to obtain ∼10^8^ CFU/mL (OD_600nm_–0.08–0.10). Each yeast cell suspension (4 mL) was mixed for 15 s using a vortex mixer (VM-10, WiseMix®, Germany) and incubated at 25 °C for 24 h without agitation. For measurement, 0.2 mL of the upper suspension was transferred to a new disposable plastic cuvette and mixed with 1.8 mL of sterile 0.9% NaCl solution and the absorbance_600nm_ was measured at 0, 2, 4 and 24 h. The auto-aggregation rate was calculated using equation [2] from Gil-Rodríguez et al. (2015).

[2] $$\text{Auto}-\text{aggregation}\;\left(\%\right)=\left[\frac{A_{0}-A_{T}}{A_{0}}\right]*100\%$$

A_T_: Absorbance_600nm_ at each time interval
A_0_: Absorbance_600nm_ at start

Co-aggregation of yeast strains with pathogenic bacteria was analysed using the method of Xu et al. (2009) and Lara-Hidalgo et al. (2019) with modifications. Yeast cells were cultured as previously described. Pathogenic strains comprising *Pseudomonas aeruginosa* MUA26*, Staphylococcus aureus* MCTIC 4163*, Escherichia coli* NCTIC 8196, *Bacillus cereus* MU-A44 were used in this study*. B. cereus* MU-A44 was cultured in Muller-Hinton broth (Thermofisher, Waltham, MA, USA) at 30 °C and all the other pathogens were cultured separately at 37 °C overnight. Both yeast and the pathogens were centrifuged at 8000 *g* for 5 min at 4 °C, washed twice and resuspended in 0.9% NaCl solution to obtain ∼10^8^ CFU/mL. Equal volumes of yeast cell suspension (2 mL) and each pathogenic bacterium were mixed for 10 s using a vortex mixer and incubated at 25 °C for 4 h without agitation The absorbance_600nm_ of the mixture was measured after incubation. Cell suspensions of individual strains were used as controls. The co-aggregation rate was calculated using equation (3) developed by Handley et al. (1987).

[3] $$\text{Co}-\text{aggregation}\;\left(\%\right)=\left(\frac{\left(A_{pro}+A_{path}\right)/2-A_{mix}}{\left(A_{pro}+A_{path}\right)/2}\right)*100\%$$

A_pro_: absorbance of control tubes of probiotic strains
A_path_: absorbance of control tubes of pathogenic bacteria
A_mix_: absorbance of control tubes of mixture after incubation for 4 h

#### 2.3.2 Cell surface hydrophobicity

The hydrophobicity properties of the yeast cells were determined according to Liu et al. (2021) and Xu et al. (2009). The yeast cells were cultured at 25 °C overnight and centrifuged at 8000 *g* for 5 min at 4 °C. The cell pellet was washed twice with sterile 0.9% NaCl and resuspended in the same buffer to achieve an OD_600nm_ of approximately 0.4–0.6 (A_0_). The adjusted yeast cell suspension (3 mL) was mixed thoroughly with chloroform (1 mL, Sigma Aldrich, St. Louis, MO, USA) using a vortex mixer for 30 s. The mixture was then incubated at ambient temperature (∼22 °C) for 30 min to separate the organic and aqueous phases. The aqueous phase was measured at OD_600nm_. The hydrophobicity was calculated as shown in equation [4] according to Xu et al. (2009).

[4] $$\text{Hydrophobicity}\;\left(\%\right)=\left(\;1-\frac{A_{X}}{A_{0}}\right)*100\%$$

A_X_: absorbance of the aqueous phase
A_0_: absorbance of the initial probiotic strains

### 2.4 Potential health promoting properties of isolated yeast strains

#### 2.4.1 Antioxidant activity

The reduction (%) of the 1,1-diphenyl-2-picrylhydrazyl (DPPH) radical was used to evaluate the antioxidant activity of the yeast isolates (Gil-Rodríguez et al., 2015; Hsu and Chou, 2021; Merchán et al., 2020). Yeast strains were cultured separately in YPD broth overnight at 25 °C. The cell pellets were harvested by centrifugation (8000 *g,* 5 min, 4 °C) and filtered through 0.22 μm syringe filters to prepare cell free supernatants (CFS). The initial concentration of the different yeast cultures (CFS) was standardised to OD_600nm_ = 1.2. The cell suspension (0.8 mL) was then mixed with 1 mL of 0.2 mM methanolic DPPH solution (Sigma Aldrich, St. Louis, MO, USA) in 2 mL microcentrifuge tubes. The solution was mixed using a vortex mixer for 1 min and then incubated for 30 min at room temperature (∼22 °C) in the dark. The reaction tubes were centrifuged at 12000 *g* for 5 min, and the absorbance of the supernatant was measured at 517 nm. The radical scavenging activity of DPPH was calculated using equation [5] according to Hsu and Chou (2021).

[5] $$Scavenging\;ability\;\%=\left[1-\frac{A_{517sample}}{A_{517\;blank\;}}\right]*100\%$$

A_517sample:_ Absorbance of sample at 517 nm
A_blank_: Absorbance of blank (methanol) at 517 nm

The Trolox equivalent antioxidant capacity (TEAC) of each yeast culture was determined using a solution of 6-hydroxy-2,5,7.8-tetramethylchroman-2-carboxylic acid (Trolox) (Sigma Aldrich, St. Louis, MO, USA) dissolved in methanol (1 mM). The Trolox was diluted with methanol to different concentrations (10–100 μM), and 0.8 mL of each concentration of Trolox was mixed with 1 mL of 0.2 mM DPPH solution and incubated at ambient temperature (∼22 °C) for 30 min in the dark. The absorbance of the mixture was measured at 517 nm using a spectrophotometer. The scavenging ability of the yeast cultures and Trolox on DPPH were measured at the same time. Therefore, the linear regression equation [6] of Trolox concentration and scavenging ability in the range of 10–100 μmol Trolox/mL was used to quantify TEAC.

[6] y = 0.9799x +11.186 R^2^ = 0.998

x: Trolox concentration (μM)
y: DPPH free radical scavenging activity (%)
R^2^: Coefficient of determination

#### 2.4.2 Bile salt hydrolase (BSH) activity

Bile salt hydrolase activity of the yeast isolates was assessed using the method of Gebre et al. (2023) with some modifications. The yeast isolates were cultured in YPD broth overnight and adjusted to 0.5 McFarland standard (∼1.5–2.0 x10^8^ CFU/mL) (Menezes et al., 2020). Ten (10) μL of standardised yeast culture were spotted on PDA plates containing 0.5% (w/v) bile salt and 0.37 g/L calcium chloride. The plates were incubated at 25 °C for 5 days. The presence of an opaque precipitation indicated a positive reaction, and the diameter of the precipitation was measured. A larger diameter indicated stronger BSH activity by the yeast isolates.

#### 2.4.3 Antimicrobial activity

The well-diffusion agar method was used to determine the antimicrobial activity of yeast isolates (Merchán et al., 2020; Srinivas et al., 2017). Four bacterial strains (*P. aeruginosa* MUA26*, S. aureus* MCTIC 4163*, E. coli* NCTIC 8196, *B. cereus* MU-A44) and two mycotoxigenic fungi (*Aspergillius brassilliensis* NZRM2578, and *Penicillium chrysogenum* NZRM2999) were used as the test microbial pathogens.

For the antibacterial activity, the yeast isolates were cultured overnight in YPD broth at 25 °C. The yeast cell density was standardised to 0.5 McFarland standard. All the pathogens were cultured in tryptone soya broth (Thermofisher, Waltham, MA, USA) and incubated at 37 °C for 24 h and adjusted to a turbidity equivalent to 0.08–0.10 at OD_600nm_. About 50 μL of pre-incubated pathogens (∼0.5 McFarland standard) were spread onto solidified Muller-Hinton Agar (MHA) plates (Sigma Aldrich, St. Louis, MO, USA) with sterile cotton swab and wells (Ø ≈ 7 mm) were made with a sterile stainless steel cork borer. Standardised yeast culture (100 μL) was added to the wells of the MHA plates and incubated at 37 °C for 24 h. The diameter of clear zones was measured to determine the magnitude of antibacterial activity.

The antifungal activity of yeast isolates was evaluated with the method of Merchán et al. (2020); Rodríguez-Tudela et al. (2001) with slight modifications. The fungi were inoculated on PDA plates at 25 °C for 5 days until sporulation. The fungi culture suspension was adjusted to 0.5 McFarland standard at OD_530nm_ (∼0.11–0.14) containing approximately 10^5^ cfu/mL (Rodríguez-Tudela et al., 2001). About 50 μL of pre-incubated fungi were spread on the PDA plates with sterile cotton swab. Wells (Ø ≈ 7 mm) were made with a sterile stainless steel cork borer. Standardised yeast culture (100 μL) was added to the wells in the PDA plates and incubated at 25 °C for five days. The diameter of clear zone around each disk was measured to determine antifungal activity.

### 2.5 Safety assessment

#### 2.5.1 Susceptibility to antibiotics

Disk diffusion was used to determine the antibiotic susceptibility of yeast isolates (Fernández-Pacheco et al., 2021; Goktas et al., 2021; Liu et al., 2021). Antibiotic resistance was determined using the Mast-ring S® composed of eight clinical antibiotics (μg) as follows: ampicillin 25, chloramphenicol 50, colistin sulphate 100, kanamycin 30, nalidixic acid 30, nitrofurantoin 50, streptomycin 25, and tetracycline 100. The yeast strains were cultured in YPD broth overnight at 25 °C. Then, 50 μL of each yeast suspension (0.08–0.10 at OD_600nm_) was evenly spread on the PDA plates. A Mast-ring S ® disk was gently placed on the surface of each PDA plate with sterile forceps. The diameter of clear zone around each disk was measured after 5 days incubation at 25 °C.

#### 2.5.2 Haemolytic activity

The haemolytic activity of the yeast strains was performed using the method of Menezes et al. (2020) and Fernández-Pacheco et al. (2021). A loopful of fresh yeast culture was streaked on blood agar plates (5% sheep blood) and incubated at 25 °C for 5 days. The presence of a green or brown zone around colonies indicated α-haemolysis. The development of a clear zone around colonies was referred to as β-haemolysis. If no clear zone developed around the colonies this was considered as γ-haemolysis. *S. aureus* was used as a positive control.

#### 2.5.3 Proteolytic activity

The proteolytic activity of yeast isolates was determined according to the method of Moslehishad et al. (2013) and Gut et al. (2019). A suspension (∼10 μL) of activated yeast culture was spot-inoculated on nutrient agar plates containing 5% (w/v) skim milk (Anchor, New Zealand). Plates were incubated at 25 °C for 5 days. The presence of a clear zone around the colony indicated a positive reaction, and the diameter of the clear zone was measured. The size of the diameter of the clear zone around the colony was indicative of the strength of the proteolytic activity of the yeast isolates.

#### 2.5.4 Phospholipase activity

Phospholipase production was evaluated on egg yolk medium (Price et al., 1982). The medium consisted of PDA supplemented with 5.85% (w/v) NaCl, 0.05% (w/v) CaCl_2_ and 5% sterile egg yolk. A suspension (10 μL) of overnight yeast culture (∼10^6^ CFU/mL) was inoculated onto the agar plates and incubated at 25 °C for 5 days. Enzymatic activity was visualised as areas of precipitation around each yeast colony. Phospholipase activity (P_z_) was expressed as the ratio of the colony diameter to the total diameter of colony and precipitation. Hence, the P_z_ = 1 indicated negative activity, lower P_z_ values indicated higher enzymatic activity.

#### 2.5.5 Gelatinase activity

The gelatinase activity of yeast isolates was carried out using the stabbing method described by Pereira et al. (2021). Fresh yeast culture was stabbed into nutrient gelatine medium and incubated at 25 °C for up to 14 days and placed at 4 °C for 30 min to check for liquefaction. The presence of liquefaction was considered as a positive reaction.

### 2.6 Statistical analysis

All the experiments were performed in triplicate. Percentages, means, and standard error of means were analysed by Microsoft Excel (Microsoft, USA). Statistical analysis of data was carried out by analysis of variance-one way (ANOVA-ONEWAY) using Minitab 21 (Minitab, USA) to compare the means (p < 0.05). Significant differences between the means were separated using Tukey's test. Correlation analysis of Pearson correlation coefficient and principal component analysis (PCA) of probiotic characteristics were performed using Origin® 2021b (OriginLab, Northampton, USA).

## 3 Results and discussion

Based on information from the literature, several of the strains (*D. bruxellensis* (LBY1), *B. anomalus* (LBY5), *S. pombe* (LBY5) and *H. valbyensis* (DOY1)) investigated in the current study are likely to contribute to the appealing flavour, aroma, and taste of fermented beverages (Fadda et al., 2001; Mayser et al., 1995; Wang et al., 2022). *P. kudriavzevii* (GBY1) is known to produce a biofilm and flavour compounds in kombucha, while yeast strains of *D. prosopidis* (D3Y9, D5Y12, D7Y18) are associated with the sensory profile of kombucha (Chelliah et al., 2016; Wang et al., 2023). Although strains of *Z. lentus* (D7T19, D11Y30, D14Y35, TFY36, TFY38, TFY39) have been reported as food spoilage yeast (Steels et al., 1999; Marsh et al., 2014), they are likely to have a positive contribution on the flavour of the fermented beverage (Wang et al., 2023). *S. cerevisiae* (GBY2) has been widely used for fermentation and is well-recognised as being safe for human consumption (Wang et al., 2022). However, safety studies do not appear to have been conducted on the other strains. Therefore, both the probiotic characteristics and safety assessments of these fifteen isolates were investigated here.

### *3.1* *In vitro* tolerance to simulated gastrointestinal tract (GIT) conditions

#### 3.1.1 Growth tolerance at different temperatures

Growth or tolerance at different temperatures, low pH, in the presence of bile salts and sodium chloride solutions are considered as a prerequisite for screening probiotic strains which will likely survive in the human gut (Menezes et al., 2020) (Table 2). All the yeast strains (n = 15) were able to grow at 25 °C. Of the strains, three isolates (20%) which belonged to *D. prosopidis* showed weak growth at 37 °C, and six *Z. lentus* isolates (40%) were not able to grow at above 30 °C. The remaining six strains were able to grow well at 37 °C; of these, five strains which belonged to *D. bruxellensis, S. pombe, P. kudraivzevii,* or *S. cerevisiae* were also able to grow well at 39 and 42 °C. However, the *H. valbyensis* DOY1*,* was unable to grow at either of the two highest temperatures tested (Table 2).

**Table 2:** Growth of yeast strains at different temperatures and in different NaCl concentrations.

Isolates and identities	Growth temperature (°C)					Growth in NaCl_aq_ (w/v)		
	25	30	37	39	42	2.5%	4%	6.5%
*Debaryomyces prosopidis* D3Y9	+	+	w	–	–	+	+	+
*Debaryomyces prosopidis* D5Y12	+	+	w	–	–	+	+	+
*Debaryomyces prosopidis* D7Y18	+	+	w	–	–	+	+	+
*Zygosaccharomyces lentus* D7Y19	+	–	–	–	–	+	+	+
*Zygosaccharomyces lentus* D11Y30	+	–	–	–	–	+	+	+
*Zygosaccharomyces lentus* D14Y35	+	–	–	–	–	+	+	+
*Zygosaccharomyces lentus* TFY36	+	–	–	–	–	+	+	+
*Zygosaccharomyces lentus* TFY38	+	–	–	–	–	+	+	+
*Zygosaccharomyces lentus* TFY39	+	–	–	–	–	+	+	+
*Dekkera bruxellensis* LBY1	+	+	+	+	+	+	+	–
*Sachizosaccharomyces pombe* LBY5	+	+	+	+	+	+	+	w
*Hanseniaspora valbyensis* DOY1	+	+	+	–	–	+	+	+
*Brettanomyces anomalus* DOY8	+	+	+	+	+	+	+	+
*Pichia kudraivzevii* GBY1	+	+	+	+	+	+	+	+
*Saccharomyces cerevisiae* GBY2	+	+	+	+	+	+	+	+

Note: “+” indicated positive results; “-” indicated the negative results; “w” indicated weak growth’.

Some non-*Saccharomyces* yeasts such as *Z. lentus* can grow at low temperatures (Steels et al., 1999), possibly due to their lipid composition (Alvarez et al., 2023). Kombucha is commonly fermented between 25 and 30 °C for 7–14 days (Leonarski et al., 2022), hence, the ability to grow at temperatures below 30 °C is recommended for cultures intended for use in kombucha production. Growth at 37 °C (normal human body temperature) is an important criterion for probiotic selection (Alvarez et al., 2023; Gil-Rodríguez et al., 2015; Hsu and Chou, 2021; Menezes et al., 2020), however, it is also important to test the tolerance of potential probiotic yeast at higher temperatures of between 39 °C (febrile state) and 42 °C (hyperpyrexia-related to bacterial infection) (Alvarez et al., 2023; de Llanos et al., 2006). In the current study, five strains (LBY1, LBY5, DOY8, GBY1, and GBY2) showed good growth at five different temperatures (25, 30, 37, 39 and 42 °C) which indicated that the temperature tolerance of these strains may improve their survival during the heat treatment such as spray drying (Desmond et al., 2002).

#### 3.1.2 Tolerance to different NaCl concentrations

All the yeast isolates showed good tolerance in 2.5%–4% NaCl (w/v) **(**Table 2**).** Strains of *D. bruxellensis* were not able to grow at 6.5% NaCl (w/v) whereas *S. pombe* LBY5 showed weak growth along the streaked lines at this salt concentration. Some probiotics have been isolated from savoury fermented foods containing NaCl including fermented black olives, fish, and meat (Tamang and Lama, 2022). The addition of salt in fermented food products not only contributes to the aromatic profile but salt is also added as a preservative to inhibit the growth of pathogenic microorganisms (Alkalbani et al., 2022a,b; Zeng et al., 2019). Therefore, good growth in the presence of salt is an important and desirable characteristic of probiotics in fermented food products (Szutowska and Gwiazdowska, 2021). Most yeast strains (86.67%) in this study were able to grow in all the salt concentrations tested (2%–6.5% w/v), which may support their application in salty fermented food products (Diguță et al., 2023).

#### 3.1.3 Tolerance to low pH

**Fig. 1:**
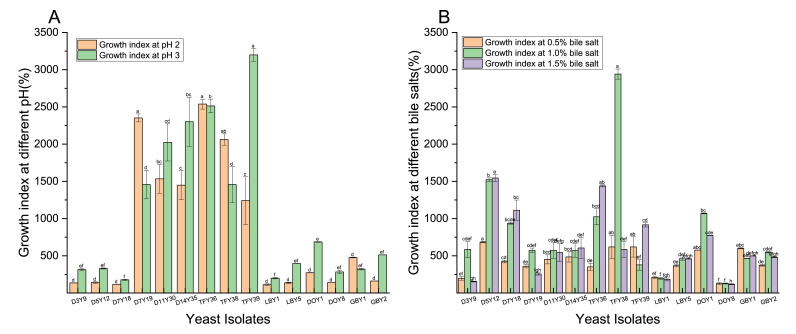
Growth index of yeast strains at low pH (A) and in the presence of bile salts (B). Different lowercase letters (a–f) above the chart bars indicate significant differences (p < 0.05); n = 3 triplicate experiments; error bars indicate SD of means.

Another important challenge for probiotic yeast isolates is tolerance to extreme high acidic conditions (pH 2–3) in the stomach (Alkalbani et al., 2022a,b; Pereira et al., 2012). Therefore, a fundamental selection property of probiotic yeast is that they tolerate low pH to survive in the gastric juice (Pereira et al., 2012). All 15 yeast isolates in this study exhibited excellent growth at pH 2 and 3 (p < 0.05) (Fig. 1A), indicating their ability to survive in simulated gastric juice *in vitro*. Most strains (73.33%) had a higher tolerance at pH 3 than pH 2 under similar incubation conditions. Four isolates (D7Y19, TFY36, TFY38, GBY1) exhibited better tolerance at pH 2 than pH 3, which was similar to the pH tolerance of *P. kudraivzevii* reported by Alvarez et al. (2023). Hsu and Chou (2021) reported that *D. bruxellensis, P. kudraivzevii* and *S. cerevisiae* yeast isolated from kombucha, fermented vinegar and milk kefir produced in Taiwan also showed good growth at pH 3 which was consistent with our results. The tolerance to low pH conditions was not surprising as this level of acidity is commonly found in kombucha and other fermented beverage products such as cider or kefir (Goktas et al., 2021; Tran et al., 2020). Based on the results, all 15 strains tested in the present study were considered as acid tolerant.

#### 3.1.4 Tolerance to bile salts

In addition to tolerance under high acid environments, another major barrier for probiotics to survive in the intestinal tract is the high bile salt concentration (Alkalbani et al., 2022a,b). Bile salts are lipid emulsifying agents released in the duodenum after food ingestion, bile salts also have antimicrobial activity (Urdaneta and Casadesús, 2017). All 15 yeast strains tested here exhibited excellent growth after 24 h incubation in the presence of 0.5%–1.5% (w/v) bile salts (Fig. 1B). However, the growth of the microorganisms in the presence of different bile salts concentrations was strain-dependent (p < 0.05), with the highest growth rate observed for D5Y12 in 1.0% (w/v) bile salts, while the lowest growth was for DOY8 in 1.5% (w/v) bile salts. Although others have shown the growth rate of certain probiotic strains increase at lower bile salt concentrations, no such correlations were observed in the current study (Topçu et al., 2020).

### 3.2 Cell surface characteristics

The ability of potential probiotics to adhere to epithelial cells and mucosal surfaces is an important cell surface characteristic because it allows probiotics to persist longer in the GI tract, thus allowing more time for interactions to occur with host epithelial cells and hence greater potential to confer their health benefits to the host (García-Cayuela et al., 2014; Pereira et al., 2012; Suvarna et al., 2018). The adhesion properties of probiotics are complex, involving electrostatic and van der Waals forces between the cell surface of the probiotic and intestinal cells, especially the interaction between the physical and chemical composition of the probiotic cell surface and intestinal cell (Alvarez et al., 2023; Menezes et al., 2020). The auto-aggregation and hydrophobicity properties of probiotic yeast are related to their ability to adhere to the intestinal mucosa (Hsu and Chou, 2021; Pereira et al., 2012). In addition, the ability of probiotics to co-aggregate with pathogenic bacteria is a defense mechanism to inhibit the colonization of pathogens in the intestine (Liu et al., 2021; Menezes et al., 2020).

#### 3.2.1 Auto-aggregation activity

**Fig. 2:**
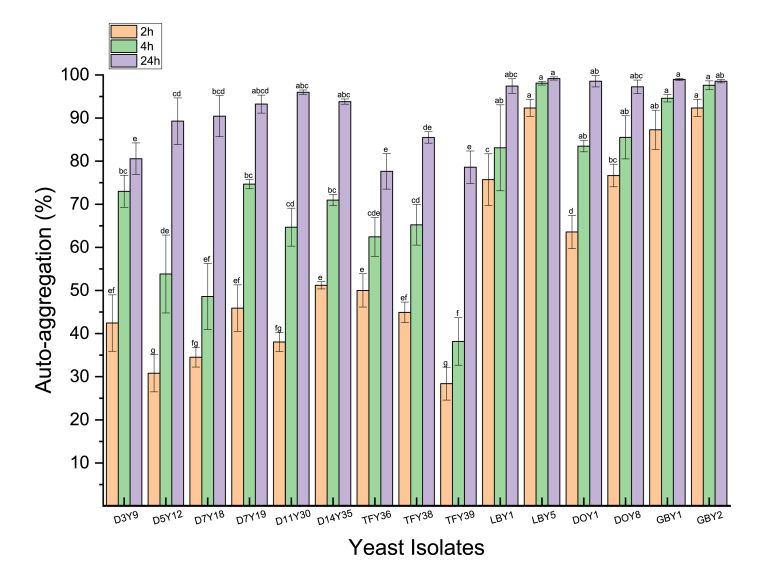
Auto-aggregation (%) of yeast strains after 2, 4 and 24 h incubation. Different lowercase letters (a–g) above the chart bars indicate significant difference (p < 0.05); n = 3 triplicate experiments; error bars indicate SD of means.

Auto-aggregation is the reversible adherence between identical cells resulting in a precipitate (Hsu and Chou, 2021; Trunk et al., 2018). This characteristic protects probiotic yeast from extreme conditions such as oxidative stress and nutrient deficiency (Fekri et al., 2020; Trunk et al., 2018). Auto-aggregation can also improve the adherence of probiotics to intestinal cells and prevent pathogens from colonising the GI tract (García-Cayuela et al., 2014). Fig. 2 shows the auto-aggregation of the 15 yeast isolates. The yeast strains exhibited marked variability in their level of auto-aggregation after incubation for 2 h (p < 0.05), ranging from 28.39 ± 3.81% (TF39) to 92.35 ± 0.87% (LBY5). Six strains (LBY1, LBY5, DOY1, DOY8, GBY1 and GBY2) showed a high percentage (>60%) of auto-aggregation after 2 h incubation. Of these two yeast strains, *S. pombe* (LBY5) and *S. cerevisiae* (GBY2) showed very high auto-aggregation (>90%), forming an obvious precipitate at the bottom of the tubes after 2 h incubation. The remaining strains showed auto-aggregation levels of 20%–60% after 2 h. Most strains (80%) showed increased auto-aggregation (62.44%–97.61%), between 2 and 4 h incubation (Fig. 2). With the exception of TFY36 and TF39 (both belonging to *Z. lentus*), all yeast tested in this study showed greater than 80% auto-aggregation after 24 h incubation. In this study, *S. pombe* LBY5 showed the fasted and best auto-aggregation performance of the 15 strains tested. Alvarez et al. (2023) evaluated auto-aggregation of five *P. kudraivzevii* yeast strains and showed much lower auto-aggregation percentages at 4 h inoculation than our study. However, after 24 h incubation, their results were similar to ours (98.54 ± 0.25 %). As expected, *P. kudraivzevii* exhibited better auto-aggregation performance than bacteria, as yeast cells are usually heavier and larger. Accordingly, they easily precipitate in higher proportions (García-Cayuela et al., 2014; Hsu and Chou, 2021). The auto-aggregation capacity of yeast isolates can be affected by their different cell wall constituents, appendages (fimbriae), adhesins, and macromolecules (Nwoko and Okeke, 2021; Touhami et al., 2003). Hence, this may explain the variation of auto-aggregation capacity of yeast strains in this study.

#### 3.2.2 Co-aggregation activity

**Fig. 3:**
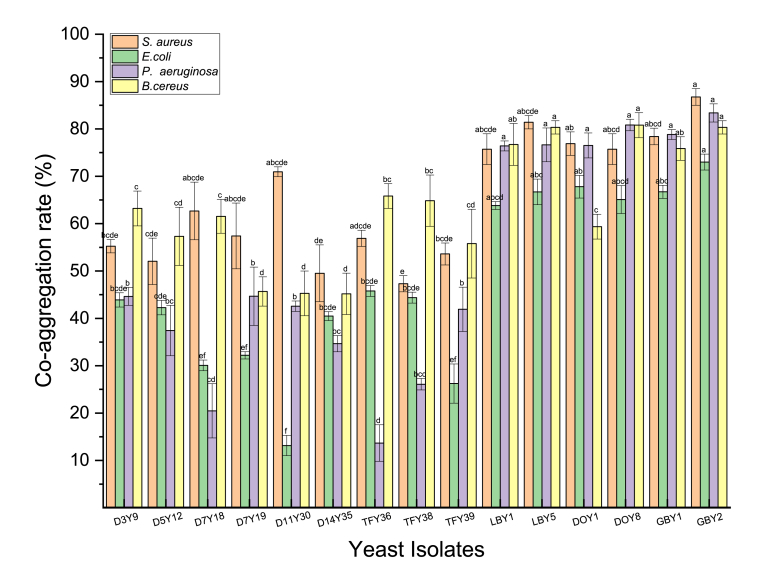
Co-aggregation ability of yeast strains with pathogenic bacteria. Different lowercase letters (a–f) above the chart bars indicate significant difference (p < 0.05); n = 3 triplicate experiments; error bars indicate SD of means.

In contrast to auto-aggregation, co-aggregation occurs between genetically different microbial strains (Collado et al., 2008). All the yeast strains tested here exhibited different co-aggregation capacities against four pathogenic bacteria (two Gram-positive and two Gram-negative). Among the yeast strains, *S. cerevisiae* GBY2 showed the highest co-aggregation (%) against *S. aureus*. High co-aggregation rates (>80%) were also recorded for LBY5, GBY2, and DOY8 against *B. cereus*. These three yeast strains also showed high co-aggregation which is likely to be linked to auto-aggregation activity (Vlková et al., 2008). The lowest co-aggregation capacity was recorded for *Z. lentus* D11Y30 against *E. coli* (Fig. 3). All the yeast strains tested exhibited higher co-aggregation rates against Gram-positive (*S. aureus* and *B. cereus*) than against Gram-negative pathogens (*P. aeruginosa* and *E. coli*). Results obtained in this study suggested that the co-aggregation capacity was dependent on the incubation conditions, specific combinations of pathogenic bacteria and yeast strains which is consistent with results from previous studies (Lara-Hidalgo et al., 2019). The high co-aggregation rate against the four pathogens tested allows the yeast cells to develop a competitive microenvironment around the pathogens, displacing them and preventing pathogens from adhering to host epithelial cells. The displacement of pathogens protects the host against pathogenic infection (Burns et al., 2011). Therefore, the high co-aggregation capacity of yeast isolates in the present study with the four tested pathogens indicated that these yeast strains may be beneficial for host health.

#### 3.2.3 Hydrophobicity activity

**Fig. 4:**
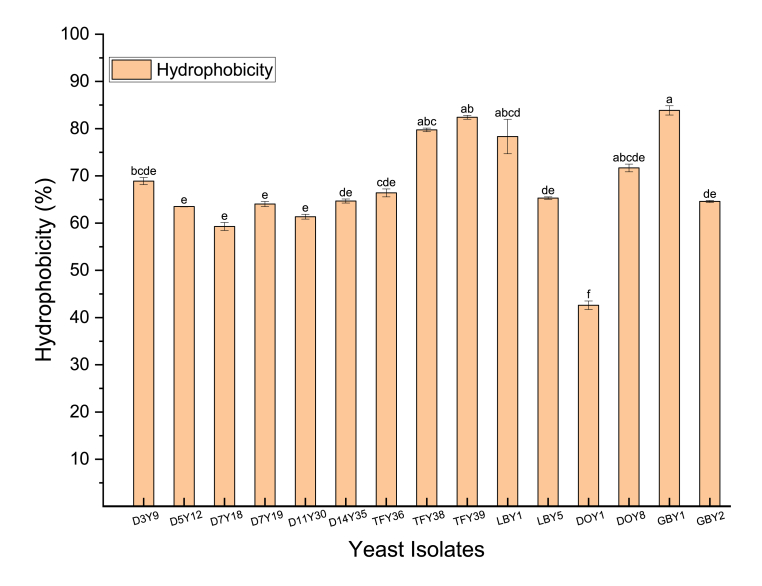
Hydrophobicity (%) of yeast strains. Different lowercase letters (a–e) above the chart bars indicate the significant difference (p < 0.05); n = 3 triplicate experiments; error bars indicate SD of means.

Modified adhesion ability to hydrocarbons (MATH) has been widely used for evaluating cell surface hydrophobicity of probiotic microbes (Chelliah et al., 2016; García-Cayuela et al., 2014; Suvarna et al., 2018). In this study, cell surface hydrophobicity was evaluated by affinity to chloroform (polar acid solvent) (Liu et al., 2021). According to Lara-Hidalgo et al. (2019), hydrophobicity rates higher than 40% indicate the yeast isolates are hydrophobic. The yeast strains tested exhibited moderate to high hydrophobicity in chloroform, varying from 42.63 ± 0.91% to 83.87 ± 0.98% (Fig. 4) with a significant difference between the 15 strains (p < 0.05). Of the 15 yeast strains in the present study, *P. kudraivzevii* GBY1 exhibited the highest hydrophobicity (83.87 ± 0.98%) to chloroform which was consistent with the results obtained by Helmy et al. (2019). This strain also had the highest auto-aggregation percentage. Results showed that hydrophobicity properties varied between strains and species probably due to the different composition of growth media, age of microorganisms, or surface-associated proteins between the strains (García-Cayuela et al., 2014; Nwanyanwu et al., 2012). Cell surface hydrophobicity is important in both proliferation and adhesion of probiotics (Sourabh et al., 2011). Having a high cell surface hydrophobicity capacity is vital as it may facilitate yeast strains to strongly attach (adhere) to hydrophobic surfaces such as the human gastric mucosa and colon through which they may confer their health-promoting effects to the host (Lugea et al., 2000).

**Fig. 5:**
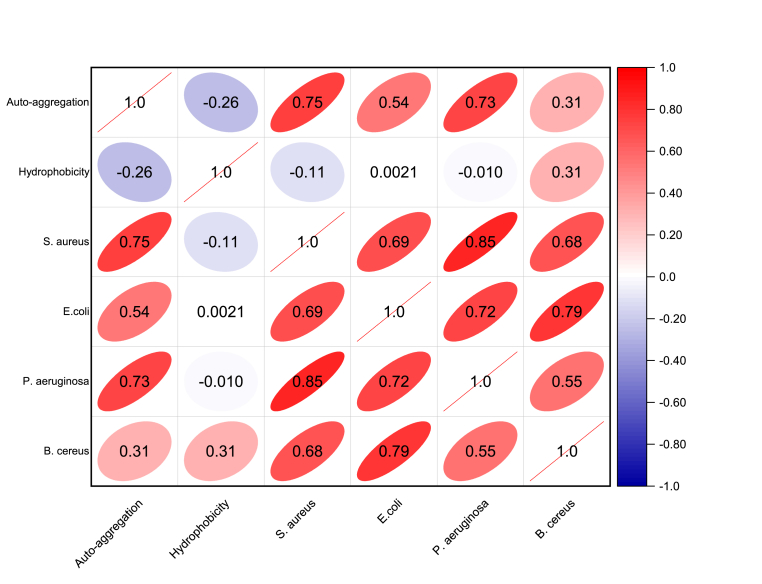
Pearson's correlation coefficient matrices between auto-aggregation, hydrophobicity, and co-aggregation (with pathogenic bacteria strains) of 15 yeast strains. *S. aureus:* yeast strains co-aggregated with *S. aureus*; *E. coli*: yeast strains co-aggregated with *E. coli*; *P. aeruginosa*: yeast strains co-aggregated with *P. aeruginosa*; Co–*B. cereus*: yeast strains co-aggregated with *B. cereus.*

Correlation coefficients (r) between auto-aggregation, hydrophobicity and co-aggregation with pathogenic bacteria are shown in Fig. 5. The auto-aggregation capacity of yeast isolates was positively correlated with their respective co-aggregation with all four pathogens, although co-aggregation was highly dependent on the combination with specific pathogenic strain of bacteria (r = 0.31–0.75). All the yeast strains in this study were positive for hydrophobicity, auto-aggregation, and co-aggregation to different extents. For example, the highest auto-aggregation (99.19 ± 0.38%) was shown by LBY5, which also showed a high co-aggregation (81.41 ± 1.40%) with *S. aureus*. Other yeast strains (DOY1, DOY8, GBY1, GBY2) also showed high auto-aggregation rates and high co-aggregation with *S. aureus, E. coli,* and *P. aeruginosa*. However, a low correlation was found between auto-aggregation and the hydrophobicity (r = −0.26) of the tested yeast strains although GBY1 showed high values for both hydrophobicity and auto-aggregation. Similarly, hydrophobicity (affinity to chloroform) showed negative or no correlation with co-aggregation and pathogens excluding *B. cereus* (Fig. 5).

Few studies have shown a high correlation between hydrophobicity and aggregation, most likely because these properties are generally strain-based (Alvarez et al., 2023; García-Cayuela et al., 2014). One possible reason for the low correlation is that adhesion to intestinal cells involves complex multifactorial interactions initiated by contact with host tissues followed by multiple cell surface interactions (García-Cayuela et al., 2014; Reuben et al., 2020). Moreover, other factors including surface associated proteins, polysaccharides, and surface charge may also affect the adhesion of probiotics to human epithelial cells. Cell surface hydrophobicity, auto-aggregation and co-aggregation with pathogens can still be used as a tool for the preliminary selection of probiotics suitable for administration to humans and animals as they may contribute to colonization of probiotics to some extent (Collado et al., 2008). However, the impact of other factors such as cell surface charge and composition may also need to be evaluated in further studies.

### 3.3 Potential beneficial effects of yeast isolates

#### 3.3.1 Antimicrobial activity

In addition to tolerance to the GI tract environment and cell surface characteristics, probiotics are also expected to confer host-associated health benefits (Kechagia et al., 2013; Pereira et al., 2012). None of the yeast isolates tested in this study inhibited the growth of pathogenic bacteria and moulds tested as no clear inhibition halos were observed. Antimicrobial activity against pathogenic bacteria and fungi is considered as an important characteristic of probiotic strains. The antagonistic activity of yeast isolates against pathogens has been related to their ability to compete for nutrients, formation of organic acids, production of high levels of ethanol, and secretion of antimicrobial compounds such as killer toxins or mycotoxins (Golubev, 2006). However, the absence of antimicrobial activity in this study was still consistent with previous studies which also found that potential probiotic yeast isolated from cheese (*P. kudriavzevii, S. cerevisiae*) exhibited no antimicrobial activity against *E. coli* and *S. aureus* (Binetti et al., 2013).

The ability to co-aggregate with pathogens is generally considered a beneficial feature for probiotics, as aggregation represents a physical barrier that can prevent surface colonization on human tissues. However, our current data revealed no antimicrobial activities against the four tested bacterial pathogens. Consequently, co-aggregation may have a potentially negative effect of promoting pathogen adhesion to human tissues (Shruthi et al., 2022). Further study is thus required to determine the precise consequences of co-aggregation and the potential antimicrobial properties of the yeast isolates, which are normally dependent on the environmental conditions.

Kombucha has been reported to have considerable antimicrobial activity against different pathogenic microorganisms such as *E. coli, S. aureus,* and *Listeria monocytogenes* (Al-Mohammadi et al., 2021; Battikh et al., 2012, 2013). The desirable antimicrobial activity of kombucha may be due to the formation of acetic or other organic acids, or other bioactive components such as bacteriocins (produced from probiotics like LAB), proteins, ethanol, and enzymes or the phenolic components from tea (Nyiew et al., 2022; Sreeramulu et al., 2000). The yeast isolates tested in this study may not contribute to the antimicrobial activity of kombucha.

#### 3.3.2 Bile salt hydrolase (BSH) activity

**Fig. 6:**
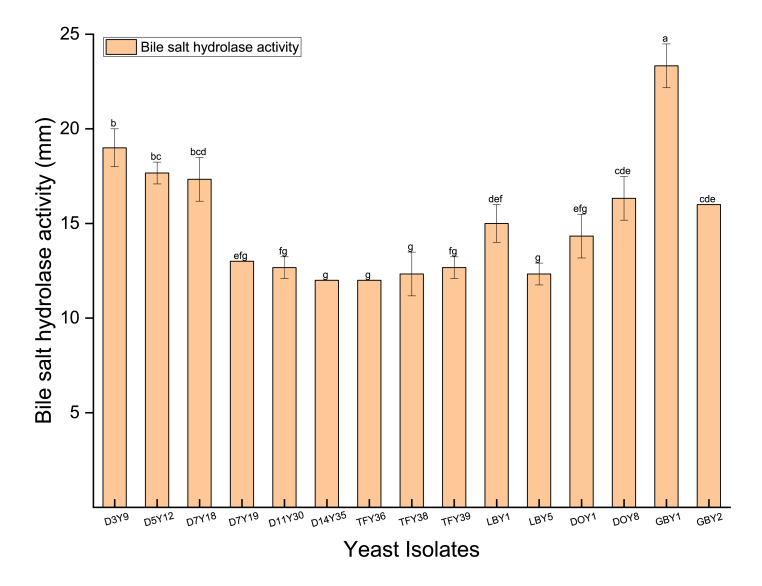
Bile salt hydrolase activity of yeast isolates. Different lowercase letters (a–g) above the chart bars indicate significant difference (p < 0.05); n = 3 triplicate experiments; error bars indicate SD of means.

The yeast isolates were inoculated on PDA plates containing differing concentrations of bile salts to determine their potential to deconjugate the bile salts. In this study, all 15 yeast strains exhibited BSH activity to different levels as indicated by the formation of a white precipitate around colonies (p < 0.05) (Fig. 6). The strongest BSH activity was observed from GBY1 with a diameter of 23.33 mm, which was consistent with *S. cerevisiae* AM18 isolated from a cereal-based fermented beverage (Gebre et al., 2023). While the weakest BSH activity was obtained from D14Y35 and TFY36 (12.00 mm). The deconjugation of bile salts by probiotic strains through bile salt hydrolase may be important for probiotic strains to adhere in the GI tract and survive in the intestine containing bile salts (Tsigkrimani et al., 2022). According to Kumar et al. (2012), a stronger BSH activity of probiotic strains may contribute to better bile salt tolerance, resulting in a higher survival rate and better colonization in the host gut. Bile salt hydrolase (BSH) activity could partially contribute to the excellent bile salt tolerance (0.5%,1.0% and 1.5%) of the 15 yeast strains in this study (Fig. 1B).

BSH is also able to break down bile salts into free primary acids (deoxycholic acid) and facilitate the intake and digestion of dietary fat (Gebre et al., 2023; Tsigkrimani et al., 2022). This characteristic may lower the risk of colon cancer and toxicity of conjugated bile salts (Noriega et al., 2006). Furthermore, BSH activity of probiotic strains has often been associated with the reduction of serum cholesterol levels, homeostasis of GI tract, and detoxification of bile, which is beneficial to the host health (Begley et al., 2005; Bommasamudram et al., 2023; Kumar et al., 2012). Hence, BSH has been considered as an alternative clinical therapy to lower blood cholesterol levels of patients with hypercholesterolemia (Chand et al., 2017). The presence of active BSH has been widely used as a primary selection criterion for potential probiotic strains in conjunction with other criteria such as safety assessments (Bommasamudram et al., 2023; Farid et al., 2021). Therefore, the BHS activity present in this study may be a useful characteristic of the yeast isolates (Begley et al., 2006).

#### 3.3.3 Antioxidant activity

**Fig. 7:**
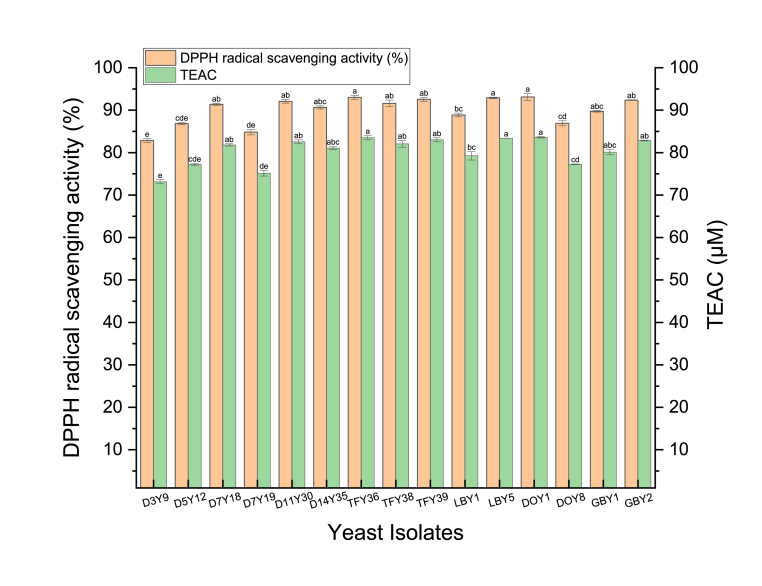
DPPH scavenging activity and TEAC of yeast strains. Different lowercase letter (a–f) above the chart bars indicated the significant difference (p < 0.05): n = 3 triplicate experiments; error bars indicate SD of means.

All the yeast strains exhibited excellent antioxidant activity with a high percentage reduction of DPPH (>80%) (Fig. 7). Six strains displayed DPPH free radical scavenging rates of between 80 and 90 % and the remainder presented DPPH rates exceeding 90%. The lowest antioxidant activity was detected from *D. prosopidis* D3Y9 (82.85 ± 0.47%) and the highest value was recorded from *H. valbyensis* DOY1 (93.13 ± 0.79%). The antioxidant activity quantified as Trolox equivalent antioxidant capacity (TEAC μM), ranged from 73.13 ± 0.48 to 83.39 ± 0.01 μM. Several studies have evaluated the antioxidant activity of yeast strains from different food samples. For example, one strain of *D. bruxellnesis* JYC2537 isolated from Taiwanese kombucha reported an antioxidant activity (>60%) which is lower than *D. bruxellensis* LBY1 (Fig. 7) in this study (Hsu and Chou, 2021). The variation in antioxidant activities of the yeast strains may be due to other active enzymes including superoxide dismutase, glutathione peroxidase and catalase, as well as exopolysaccharides, and lipids (Chen et al., 2010; Pereira et al., 2012). One study found that intact cells had higher antioxidant activity than a yeast extract, possibly due to the high concentrations of β-glucans in the intact cell walls (Chen et al., 2010). These results may explain why the antioxidant capacity of yeast isolates in this study were much higher than those reported in other studies, as our study used intact cells while other studies did not (Gil-Rodríguez et al., 2015; Hsu and Chou, 2021; Lara-Hidalgo et al., 2019; Menezes et al., 2020).

### 3.4 Principal component analysis (PCA)

**Fig. 8:**
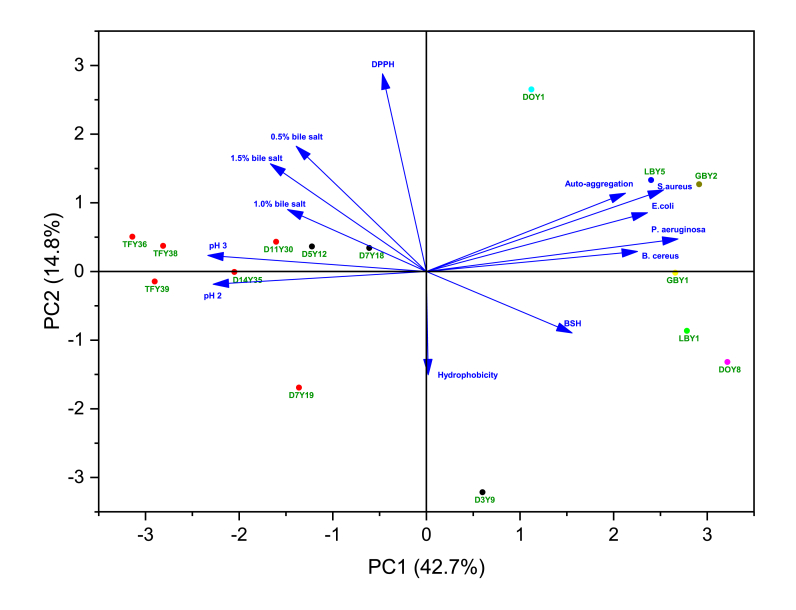
Principal component analysis (PCA) biplot of probiotic characteristics of fifteen yeast strains isolated from Kombucha. Blue arrows represent probiotic characteristics, and dots in different colours represent the yeast isolates. (For interpretation of the references to colour in this figure legend, the reader is referred to the Web version of this article.)

Multivariate principal component analysis (PCA) which focuses on dimensionality reduction, has been widely used to screen promising probiotic strains (Gao et al., 2021; Mallappa et al., 2019; Solieri et al., 2014). In this study, PCA was used to analyse the correlations between thirteen probiotic characteristics (low pH tolerance at 2 and 3, bile salt tolerance at concentration of 0.5%, 1.0%, and 1.5%, auto-aggregation, co-aggregation with four pathogens, cell surface hydrophobicity, bile salt hydrolase, antioxidant activity) and yeast strains isolated from kombucha produced in New Zealand. The results from PCA bi-plots indicated that the first two principal components explained 57.5% of the total variance in the probiotic characteristics of the fifteen yeast strains tested (Fig. 8). The principal component (PC) 1 accounted for 42.7%, and PC2 accounted for 14.8% of total thirteen variances for the yeast isolates, respectively. The PC1 revealed the maximum variations of the data and the PC2 presented the rest of variations. The fifteen yeast isolates were distributed into each of the four different quadrants. Two yeast isolates (LBY5 and GBY2) present in quadrant I (top right) showed a high correlation in auto-aggregation and co-aggregation with the four different pathogens. Six yeast strains (D5Y12, D7Y18, D11Y30, D14Y35, TFY36, TFY38) distributed in quadrant II (top left) were related to antioxidant activity and tolerance to bile salts and low pH. Auto aggregation, co-aggregation with *S. aureus*, *B. cereus, P. aeruginosa*, and *E. coli* had similar scores along PC1, which could be important characteristics to differentiate and select the most potential probiotic strains. The highest weight was obtained from antioxidant activity for the PC2. Most variables contributed positively to the PC2 except hydrophobicity, tolerance to low pH at 2, and bile salt hydrolase activity. Based on the PCA biplots and overall tolerances to temperature and NaCl, two yeast isolates (GBY1, GBY2) appear to be the most promising probiotic strains of those tested as they exhibited the highest probiotic performance and best temperature and salt tolerances.

### 3.5 Biosafety assessment

#### 3.5.1 Susceptibility to antibiotics

The safety assessment of potential probiotic strains is important because some yeast isolates may be pathogenic (Fleet and Balia, 2006). Therefore, safety assessment must be carefully evaluated for all new potential probiotic strains before application for human use (Sanders et al., 2010). A key criterion for *in vitro* safety assessment of potential probiotic strains is antibiotic resistance (Lahtinen et al., 2009). Of the strains tested, two (GBY1 and GBY5) showed resistance to all eight antibiotics tested (Table 3). Yeast strain DOY8 showed resistance to most antibiotics but was sensitive to colistin sulphate. The remaining 12 yeast strains were resistant to all the antibiotics except colistin sulphate. Resistance to ampicillin, chloramphenicol and nalidixic acid by all 15 yeast strains in this study agrees with Diguță et al. 2023. The increase in antibiotic resistance of pathogenic bacteria associated with probiotic treatment is a serious safety concern to public health (Czerucka et al., 2007). Horizontal gene transfer between bacteria and pathogenic bacteria in the GI tract can make pathogens resistant to antibiotics (Pereira et al., 2012). However, antibiotic resistance genes do not transfer from yeast to bacteria, making probiotic yeast isolates safe for administration during antibiotic treatment (Czerucka et al., 2007). Therefore, the resistance of yeast isolates to antibiotics in the present study could be a beneficial characteristic as the probiotics may survive during the antibiotic treatment and confer their beneficial effects to the host (Bacha et al., 2010). However, the yeast isolates used in this study do need to be tested against a larger number of traditional antibiotics.

**Table 3:** Susceptibility of yeast strains to antibiotics.

Yeast isolates	Diameter of clear inhibition zone (mm)							
	AM	C	K	CO	NA	NI	S	T
D3Y9	0	0	0	22.37 ± 0.55	0	0	0	0
D5Y12	0	0	0	14.50 ± 0.50	0	0	0	0
D7Y18	0	0	0	15.03 ± 0.61	0	0	0	0
D7Y19	0	0	0	15.50 ± 0.50	0	0	0	0
D11Y30	0	0	0	16.33 ± 0.58	0	0	0	0
D14Y35	0	0	0	15.33 ± 0.58	0	0	0	0
TFY36	0	0	0	17.83 ± 0.76	0	0	0	0
TFY38	0	0	0	18.50 ± 0.50	0	0	0	0
TFY39	0	0	0	16.07 ± 0.12	0	0	0	0
LBY1	0	0	0	23.00 ± 0.50	0	0	0	0
LBY5	0	0	0	9.60 ± 0.36	0	0	0	0
DOY1	0	0	0	14.00 ± 1.00	0	0	0	0
DOY8	0	0	0	27.00 ± 0.65	0	0	0	10.00 ± 1.00
GBY1	0	0	0	0	0	0	0	0
GBY5	0	0	0	0	0	0	0	0

AM: Ampicillin; C: Chloramphenicol; CO: Colistin Sulphate; K: Kanamycin; NA: Nalidixic Acid; NI: Nitrofurantoin; S: Streptomycin; T: Tetracycline. 0 = no observed inhibition zone. The results were expressed as mean ± standard deviation (SD). The antibiotic susceptibility tests were done in triplicate. R: Resistant zone diameter (<12.4 mm); I: intermediate zone diameter (12.4–17.4 mm); S: susceptible (>17.5 mm).

#### 3.5.2 Enzymatic activities

Safety aspects such as origin and possible harmful activities were also evaluated in screening for potential probiotic yeast in this study (Fernández-Pacheco et al., 2021; Menezes et al., 2020). The presence of haemolytic activity may cause the development of anaemia in the host by damaging host red blood cells (Fernández-Pacheco et al., 2021). The presence of gelatinase is another virulence factor because it degrades an appreciable number of host substrates such as collagens and fibrins. Additionally, *Enterococcus faecalis* is the main causative agent of infective endocarditis, resulting in urinary tract infection, damage to the heart valves, and high death rates of up to 20% (Baldassarri et al., 2004). The infective endocarditis is initialized by formation of vegetations on heart valves (Thurlow et al., 2010). The presence of gelatinase facilitates the development of vegetations and can lead to high death rate in endocarditis (Baldassarri et al., 2004; Johansson and Rasmussen, 2013). In addition to virulence factors, high amounts of extracellular phospholipase and proteases can promote colonization by certain pathogens and cause tissue damage to host epithelial cells (D'Eça Júnior et al., 2011). Therefore, haemolytic, protease, gelatinase, and phospholipase activities should be included as part of safety studies when investigating potential probiotic strains. None of the 15 yeast strains secreted proteases, gelatinase, or phospholipase, and no positive results were obtained for the haemolytic activity. The *in vitro* tests carried out indicated no harmful properties, but *in vivo* safety testing is required to ensure the safety of probiotics for human administration.

## 4 Conclusion

In this study, the probiotic potential of yeast strains isolated from commercial kombucha produced in New Zealand were evaluated *in vitro.* Four yeast strains of *S. pombe* LBY5, *B. anomalus* DOY8 *P. kudraivzevii* GBY1, *S. cerevisiae* GBY2 exhibited excellent tolerance to simulated GI conditions (low pH, human temperatures, and presence of bile salts), cell surface hydrophobicity, auto-aggregation, co-aggregation with pathogenic bacteria, and antioxidant activity. Results showed that all the 15 strains tested in this study can be regarded as safe for consumption based on the absence of hemolysis, proteolytic, phospholipase and gelatinase activities. Strains of *P. kudraivzevii* GBY1 and *S. cerevisiae* GBY2 showed resistance to all tested antibiotics. Thus, these two yeast strains, (*P. kudraivzevii* GBY1 and *S. cerevisiae* GBY2) may be promising novel probiotic yeast for the food industry due to their excellent antioxidant activity and cell surface characteristics. However, *in vivo* testing, to analyse the health-promoting benefits of these probiotic strains as well as their stability during storage are recommended.

## Funding

This work was financially supported by 10.13039/501100001554Massey University Doctoral Scholarship through the College of Sciences for PhD candidate Boying Wang.

## CRediT authorship contribution statement

**Boying Wang:** Conceptualization, Investigation, research design, Data curation, Writing – review & editing. **Kay Rutherfurd-Markwick:** Conceptualization, Writing – review & editing. **Ninghui Liu:** for technical support. **Xue-Xian Zhang:** Conceptualization, Writing – review & editing. **Anthony N. Mutukumira:** Project management, Conceptualization, Review & editing, Research design.

## Declaration of competing interest

Authors declare no conflict of interest.

## Data Availability

The authors are unable or have chosen not to specify which data has been used.
